# Mitochondrial Membrane Potential Predicts 4-Hour Sperm Motility

**DOI:** 10.3390/biomedicines8070196

**Published:** 2020-07-07

**Authors:** Angela Alamo, Claudia De Luca, Laura M. Mongioì, Federica Barbagallo, Rossella Cannarella, Sandro La Vignera, Aldo E. Calogero, Rosita A. Condorelli

**Affiliations:** Department of Clinical and Experimental Medicine, University of Catania, Policlinico “G. Rodolico”, via S. Sofia 78, 95123 Catania, Italy; angela.alamo1986@gmail.com (A.A.); claudiadeluca92@gmail.com (C.D.L.); lauramongioi@hotmail.it (L.M.M.); federica.barbagallo11@gmail.com (F.B.); rossella.cannarella@phd.unict.it (R.C.); sandrolavignera@unict.it (S.L.V.); acaloger@unict.it (A.E.C.)

**Keywords:** spermatozoa, total sperm motility, mitochondrial membrane potential, JC-1

## Abstract

The evaluation of conventional and biofunctional sperm parameters is of fundamental importance for assessing male reproductive function. Among these, sperm motility is one of the most important parameters. Indeed, asthenozoospermia is a frequent cause of male infertility. Sperm motility depends on mitochondrial function and the measurement of mitochondrial membrane potential (MMP) better accounts for the function of this intracellular organelle. On the basis of these premises, the present study assessed whether the MMP predicts sperm motility at 4 h in patients with low or normal MMP. To accomplish this, 31 men were enrolled. Sperm analysis was conducted according to the WHO 2010 criteria. Particular attention was paid to the evaluation of MMP after liquefaction (T0) using JC-1 staining by flow cytometry. Sperm total and progressive motility were measured at T0 and after 4 h from seminal fluid collection (T4). Patients were divided into two groups based on their sperm mitochondrial function at T0. Group A (*n* = 18) was composed of men with normal mitochondrial function since they had a percentage of spermatozoa with low MMP (L-MMP) below the normal reference value of our laboratory (<36.5%). In contrast, group B (*n* = 13) was made up of men with impaired sperm mitochondrial function (L-MMP > 36.5%). Group A had a slight but not significant reduction in total and progressive sperm motility at T4 compared with the values recorded at T0. In contrast, patients in group B showed a significant decline in both total and progressive sperm motility at T4 compared with T0 (*p* < 0.05). The results of this study showed that worse mitochondrial function, assessed by staining with JC1, is associated with a significant decline in sperm motility over time. These findings may be of clinical relevance in programs of assisted reproduction techniques. Based on our knowledge, there is no other evidence in the literature that has shown this relationship in healthy men with low MMP of idiopathic etiology, but normozoospermics according to the WHO 2010 criteria.

## 1. Introduction

Infertility is a global problem that affects about 15% of couples [1,2]. In about half of these cases, infertility is due to a male factor [3]. In clinical settings, semen quality is examined routinely and it is regarded as a crucial laboratory test for the evaluation of infertile couples [4]. Semen quality measures include sperm concentration, total sperm count, sperm motility and the evaluation of sperm morphology using the criteria established by the World Health Organization [5].

The motility classes by microscopic analysis are “progressive” for spermatozoa that move rapidly both with rectilinear motion and in large circles without considering speed, but evaluating their progression; “not progressive” for spermatozoa that move without progression, movement in situ; “absent” for immobile spermatozoa. Normal motility is defined by a percentage of spermatozoa with progressive motility greater than 32% and a percentage of total motile spermatozoa (progressive and not progressive) greater than 40%. Astenozoospermia is a condition with values lower than those indicated [5]. The percentage of motile spermatozoa decreases progressively, beginning one hour after ejaculation, at a rate of about 5% to 10%/hour. In the majority of cases, sperm velocity increases for the first 4 h and then decreases gradually [6]. In current the seminological practice, observation of sperm motility several hours after ejaculation is not considered useful. In the past, there were several reports of the usefulness of this parameter. The study of Zollner and colleagues showed that sperm motility measured after different hours of incubation positively correlated with the fertilizing ability of sperm in vitro in Spearman’s rank correlation test: motility after 0 h (*p* < 0.02), after 4 h (*p* = 0.0025) after 24 h (n.s.) and after 48 h (*p* = 0.0071) [7].

Sperm motility is certainly a fundamental property of the spermatozoon and its decrement is a frequent cause of infertility. Particularly, progressive sperm motility is related to pregnancy rate [8]. Sperm motility is the result of the propagation of waves along the flagellum in a proximal to distal direction that produces a hydrodynamic impulse, and ATP production is essential to ensure a valid motility. The formation of ATP occurs through two metabolic pathways: mitochondrial respiration (through oxidative phosphorylation) and glycolysis.

ATP synthesis is known to be more efficient in mitochondrial respiration than in glycolysis [9]. The internal mitochondrial membrane contains a multi-enzymatic complex of electron transport chains, used for mitochondrial respiration. During the passage of electrons through the chain, protons [H^+^] expelled from the mitochondrial matrix to the intermembrane space create an electrochemical proton gradient across the inner mitochondrial membrane and a return flow of H^+^ to restore the electrochemical balance. The displacement of these protons, in turn, produces a voltage gradient of about 180–200 mV, polarizing the membrane negatively towards the inside and positively outside. This voltage is defined as mitochondrial membrane potential (MMP) [10] and spermatozoa with low MMP (L-MMP) have worse sperm motility due to the lower production of ATP [8], unlike those with high MMP (H-MMP), who show better sperm quality [10].

MMP is one of the parameters that now could be considered for the complete evaluation of sperm quality besides the parameters considered in the spermiogram. However, there are also other very important parameters, such as tests of genome integrity: chromatin condensation and DNA fragmentation [11,12].

A number of factors can alter the mitochondrial function. These include diseases involving the reproductive tract (varicocele, prostatitis, etc.) [13]; some life styles (cigarette smoking, alcohol abuse, drug addiction, etc.) [14,15,16]; environmental pollution [17,18], other endocrine diseases (i.e., diabetes mellitus type 1 and 2; hyper- or hypo-thyroidism) [19,20,21] and all the causes that increase oxidative stress [22]. MMP may therefore be used as an index of sperm quality/function [8,23]. 

The importance of mitochondrial function has already been shown by other studies that, using respiratory complex inhibitors, have reported a sperm motility decrement [24,25]. Evaluation of MMP is the parameter that best reflects sperm mitochondrial function and it is an indicator of mitochondrial energy status [26]. A recent study assessed the value of MMP to predict sperm mitochondrial function [27] and some studies have shown a correlation between sperm mitochondrial function evaluated by MMP measurement and sperm motility, even after antioxidant treatments, including myoinositol, which by improving the MMP consequently also improves sperm motility [28,29]. The correlation between these parameters has also been confirmed on a large number of patients [30].

In the clinical practice, it is necessary to consider that a large number of men have apparently normal seminal parameters [31]. These men, especially if accompanied by women of advanced age, represent a critical element for therapeutic choices. On the one hand, the clinician, especially in the presence of an adequate ovarian reserve, tends to comfort the couple and suggests continuing to search for natural pregnancy, empirically adding an antioxidant treatment. On the other, the duration of the couple’s infertility and the advanced age of the woman represent prognostically unfavorable aspects. This gray area called idiopathic male infertility [31] represents, together with the primary prevention of male infertility, one of the greatest challenges of modern medical andrology. We move in the context of very delicate aspects ranging from the choice of an in vitro fertilization technique associated with a relative success rate and not indifferent economic costs to the risk of creating false expectations in the couple with significant emotional repercussions and with the aggravating circumstance of further wasting precious time.

Based on these premises, the aim of this study was to evaluate whether sperm mitochondrial function may predict sperm motility changes over time. In the present study, we evaluated if MMP predicts sperm motility at 4 h in patients with idiopathic low MMP compared with patients with normal MMP.

To accomplish this, MMP was evaluated by JC-1 staining and flow cytometry, in 31 healthy men with normozoospermia 1 h after semen collection (T0) and total and progressive sperm motility was assessed at T0 and 4 h later (T4).

## 2. Patients Selection

The study was conducted on 31 men, aged between 17 and 54 years (33.2 ± 9.5 years), attending the Division of Andrology and Endocrinology, University of Catania, for semen analysis. They had not been medically or surgically treated before entry into the study. Men with one or more of the following conditions were excluded from the study: azoospermia, FSH serum levels >8 IU/L, primary testicular diseases, (leucocyte < 1 mil/mL), central hypogonadism, systemic diseases, chronic exposure to occupational toxicants, intake of drugs, smoking or alcohol abuse and insulin resistance. Sperm analysis and flow cytometry analysis, to evaluate sperm MMP, were performed in all men at T0 and both progressive and total motility were revaluated at T4 in every man enrolled in this study. Sperm cells were in the seminal plasma through the 4 h.

The protocol was approved by the Institutional Review Board of the Division of Andrology and Endocrinology of the teaching hospital “G. Rodolico”, University of Catania (Catania, Italy), composed of M.D.M.; S.L.V.; A.E.C.; A.B.; N.B.; C.C.; C.L. (protocol number 2/3/2020) and an informed written consent was obtained from each patient.

## 3. Materials and Methods Section

### 3.1. Sperm Analysis

Sperm analysis was performed according to the WHO criteria [5]. Each seminal analysis was carried out by two operators (AA and CDL) for quality control. They were kept blind to the results of JC-1 staining before to re-evaluate total and progressive sperm motility at T4.

### 3.2. Flow Cytometry Analysis

Flow cytometry analysis was performed using the flow cytometer CytoFLEX (Beckman Coulter Life Science, Milan) equipped with two argon lasers and six total fluorescence channels (four 488 nm and two 638 nm). We used the FL1 detectors for green (525 nm), FL2 for orange (585 nm) and FL3 for red (620 nm) fluorescence; 100,000 events (low velocity) were measured for each sample and analyzed by the software CytExpert 1.2.

### 3.3. Evaluation of the Mitochondrial Membrane Potential

MMP was evaluated by a lipophilic probe 5,5′,6,6′-tetrachloro-1,1′,3,3′tetraethyl-benzimidazolylcarbocyanine iodide (JC-1, DBA s.r.l, Milan, Italy) able to selectively penetrate into mitochondria.

**Figure 1:**
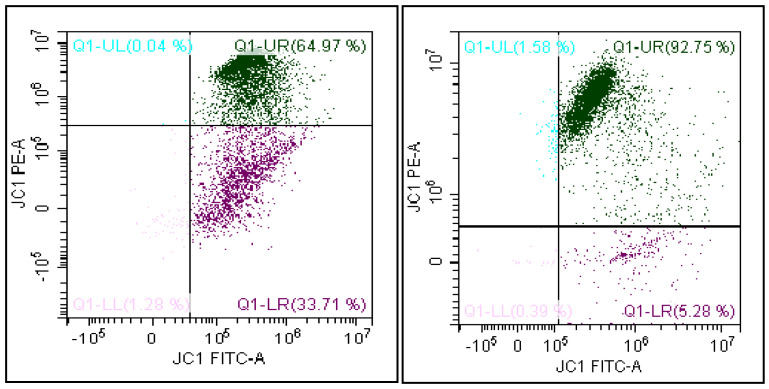
Representative dot plot of JC-1 staining. The quadrant Q1-LR shows the percentage of spermatozoa with low mitochondrial membrane potential (FITC-positive and PE-negative).

Briefly, an aliquot containing 1 × 10^6^/mL of spermatozoa was incubated with JC-1 in the dark, for 10 min, at 37 °C. At the end of the incubation period, the cells were washed in PBS and analyzed. JC-1 exists in monomeric form, emitting at 527 nm but it is able to form aggregates emitting at 590 nm. Therefore, the fluorescence changes reversibly from green to orange when the mitochondrial membrane becomes more polarized. In viable cells with normal membrane potential, JC-1 is in the mitochondrial membrane in the form of aggregates emitting in an orange fluorescence, while in the cells with low membrane potential, it remains in the cytoplasm in a monomeric form, giving a green fluorescence. Therefore, it is possible to distinguish two cell populations: cells with damaged MMP where JC-1 remains in the cytoplasm in a monomeric form, giving a green fluorescence, and cells with normal MMP with a double fluorescence where JC-1 (in addition to emitting green in the cytosol) is also in the mitochondrial membrane in the form of aggregates emitting in an orange fluorescence. The software generates a FITC/PE dot-plot that shows spermatozoa separated according to their mitochondrial membrane potential (Figure 1). In detail, the region of the dot-plot Q1-UR represents spermatozoa with high mitochondrial membrane potential (FITC- and PE-positive). The Q1-LR region, instead, represents spermatozoa with low MMP (FITC-positive and PE-negative). The latter is the value considered for data analysis in this study.

### 3.4. Statistical Analysis

Data were encoded on an Excel worksheet and transferred to a database constructed using the Statistical Package for Social Sciences (SPSS) software, version 23.0. One-way analysis of variance (ANOVA) and Student *t* test were used for comparisons of data. A *p*-value lower than 0.05 was considered statistically significant. Data were expressed as mean ± standard deviation (SD).

## 4. Results

The main sperm parameters of the 31 men enrolled in this study are shown in Table 1. Seminal fluid volume, sperm concentration and total sperm count, sperm morphology and seminal fluid leukocyte concentration were well above the lower limits established by the WHO manual [5]. The patients were divided into two groups according to the percentage of spermatozoa with low MMP (L-MMP) values to T0: group A was made up of patients (*n* = 18) with a normal percentage of spermatozoa with L-MMP (<36.5%), whereas group B consisted of patients (*n* = 13) with abnormal sperm mitochondrial function (L-MMP > 36.5%). This cut-off value was chosen according to our previous study [30]. L-MMP and H-MMP values for groups A and B are shown in Table 2. 

**Figure 2:**
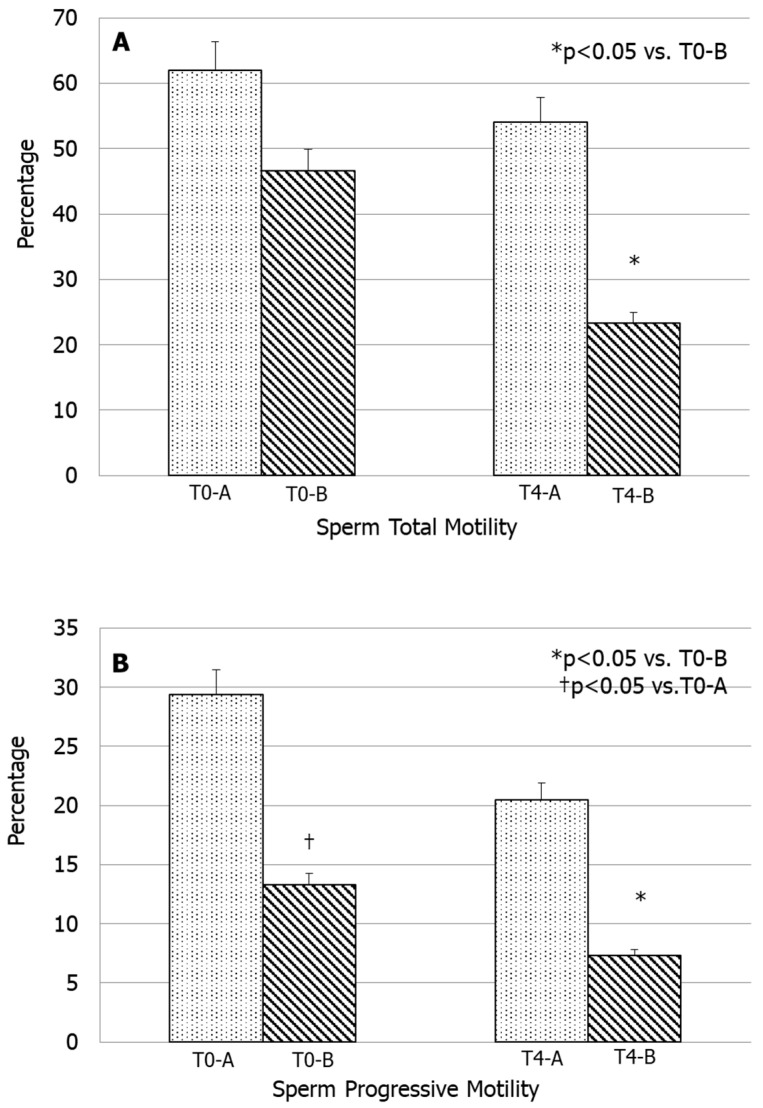
Total and progressive sperm motility of the two examined groups at T0 and T4.

**Figure 3:**
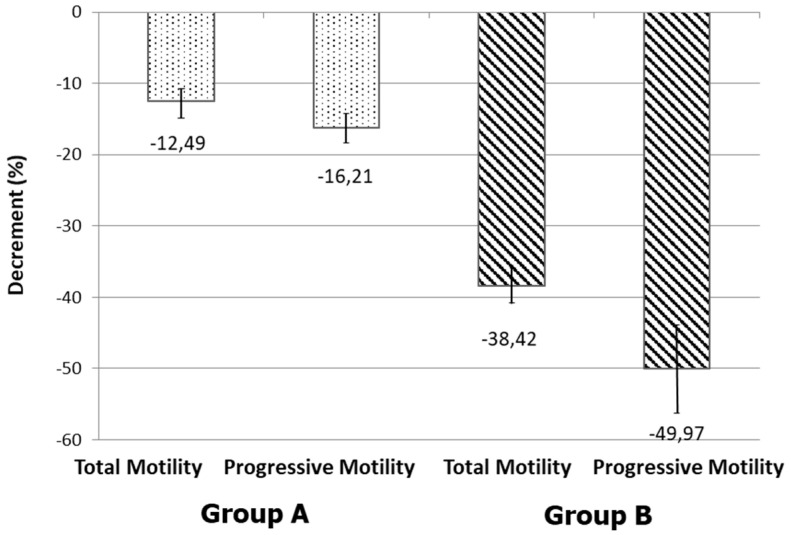
Decrease (mean of individual decrements ± standard deviation) in total and progressive motility of the two examined groups from T0 to T4.

Patients of group A with normal sperm mitochondrial function (L-MMP < 36.5% and normal percentage of spermatozoa with H-MMP) showed a slight, but not significant decrease in total and progressive sperm motility values at T4 compared with values recorded at T0. In contrast, patients of group B, with an elevated percentage of spermatozoa with L-MMP and a lower percentage of spermatozoa with normal mitochondrial function at T0, showed total and progressive sperm motility values at T4 significantly lower than that recorded at T0 (*p* < 0.05) (Figure 2A,B). In addition, total and progressive sperm motility at T4 were significantly lower in group B than those of group A (*p* < 0.05) (Figure 3). Total sperm motility decreased by −12.49 ± 4.93% (mean ± standard deviation) in group A while in group B by −38.42 ± 5.61% (Figure 2). Further, the decrement of sperm progressive motility is higher in group B (−49.97 ± 23.61) than in group A (−16.21 ± 5.30), as shown in Figure 3. 

## 5. Discussion

In patients with idiopathic sperm mitochondrial dysfunction, but apparent normal semen quality according to WHO 2010 criteria, an MMP value assessed at baseline predicts sperm motility recorded after 4 h. Based on our knowledge, there is no other evidence in the literature on this category of patients considered normozoospermic without cytometric evaluation.

The evaluation of MMP is an important marker of sperm mitochondrial function. We found that this value may predict whether sperm motility, an essential prerequisite for successful conception, will significantly deteriorate over time. The mitochondrion is responsible for supplying the energy for flagellar sperm movement. The study of mitochondrion function gives lots of important information and MMP is a parameter that closely reflects the function of this organelle. Several studies have shown a correlation between sperm motility and mitochondrial function and a recent study highlighted how the treatment with antioxidants is capable of improving MMP and consequently sperm motility, confirming the close relationship between these two parameters [28]. MMP evaluation using JC-1 was studied, for the first time, by Troiano and colleagues, who showed that it is more reliable than the previous compounds used for this purpose (i.e., rhodamine 123 staining). Therefore, it provides an accurate measurement of MMP that shows a positive correlation with sperm motility [23,32]. 

Among the healthy men with normozoospermia enrolled in the present study, ~42% (13 out of 31) (group A) had an altered MMP at baseline without any apparent reason (idiopathic form). This was associated with a significant decline of both total and progressive motility evaluated after 4 h. On the other end, the remaining 18 men who had normal sperm mitochondrial function at T0 had only a slight physiological decline of sperm motility over time.

In the 31 healthy men evaluated, both total and progressive motility were evaluated one hour after semen collection (T0) and 4 h later (T4), whereas MMP was measured at T0. According to the percentage of spermatozoa with L-MMP values (below or above 36.5%) at T0, the patients were divided into two groups. This cut-off value was chosen according to our previous study, conducted on 577 unselected men, showing that L-MMP ≤ 36.5% was associated with better semen volume, sperm concentration, total sperm count, total and progressive motility and normal forms. Moreover, this study showed that for every increase in the percentile category of sperm total and progressive motility, the risk to find an L-MMP ≤ 36.5 decreased by 1.76- and 1.27-fold, respectively [29]. The correlation between total sperm motility and MMP has already been shown [26] and Gallon and colleagues demonstrated that spermatozoa with high MMP corresponded to germ cells with high motility [33]. High values of MMP indicate the integrity of the mitochondrial structure with optimal levels of activity [10] and a high MMP is also directly correlated to a greater fertilizing capacity and a higher chance of getting good-quality embryos and an increased likelihood of achieving a pregnancy [8]. L-MMP is associated with decreased sperm motility because the flagellar movement is ATP-dependent and when the levels of ATP are low, MMP decreases [29]. 

A study, performed on the sperm motility of 33 donors, indicated that there are three typical curves of motility change during the first 4 h: increase in motility, moderate decline in motility and rapid loss of motility [6]. Sperm motility begins to fall progressively 1 h after ejaculation (above 5–10% per hour) but, in most cases, sperm velocity increases for the first 4 h and then decreases gradually. Therefore, these findings showed that there is not a unique pattern of sperm motility, especially during the first 4 h. An in vitro study on 52 infertile couples undergoing in vitro fertilization and embryo transfer, after incubation of a cumulus–oocyte complex with spermatozoa for 48 h, showed that the fertilization rate was 72.4% after 4 h for a cut-off of 60% motile spermatozoa [7]. These data showed a positive relationship between sperm motility and the fertilizing ability and a predictive power of sperm motility in the decision-making process within an assisted reproductive setting [7]. 

Our data showed that the evaluation of MMP by JC-1 staining can identify spermatozoa that will have the worst motility curve. Furthermore, MMP allowed us to identify two groups of men: those with better mitochondrial function who will therefore retain their motility over time, and those who have an altered mitochondrial function and will end up with a sharp motility decline. Therefore, this approach could help in diagnosing those men who will have a low fertilizing capability when their spermatozoa will be used several hours after ejaculation. This is particularly true in couples who undergo assisted reproductive techniques (ART).

One of the main limitations of seminology concerns the variability related to the quality and experience of the seminologist. During a regular working day, a biologist, in particular in a public center like ours, finds himself evaluating numerous samples, and this aspect can determine tiredness and loss of concentration. If we add to this the need to observe a dynamic parameter such as motility after 4 h, its commitment increases considerably. However, the evaluation of 4-hour motility expresses some functional characteristics of the spermatozoa fundamental for obtaining pregnancy. Therefore, in our opinion, having an objective parameter capable of predicting 4-hour motility is a useful tool that improves seminological practice.

Another aspect to consider concerns the physiological modulation of this cytofluorimetric parameter we are analyzing, which can be conditioned by oxidative stress or rheological alterations of the sample and which therefore suggests a real-time correction of the parameters capable of altering it. Corrections of pharmacological therapy (hormonal therapies and non-hormonal therapies) in men with apparently idiopathic infertility [31] should also consider changing the baseline value of this parameter, assuming that the specialist’s evaluation of the spermiogram after therapy takes into account progressive motility as suggested by the fifth edition of WHO 2010 [5].

We recently suggested an original therapeutic approach [31] to patients with idiopathic male infertility, identifying five possible categories: patients with an isolated increase in seminal oxidative stress markers, increase in sperm DNA fragmentation rate, infertility associated with metabolic factors, alteration of rheological features of the semen and asymptomatic men with papillomavirus DNA in the semen. In our opinion, all these categories of patients may deserve a more careful assessment of motility, without however using a method (such as 4-hour motility assessment) which is too dispendious for the time needed.

### Limitations of the Study

The study was carried out on a very small number of patients. However, it should be considered that patient selection has been very accurate. In the section of the methods, all the exclusion criteria are reported which are probably not considered routinely in clinical practice and this explains two aspects:

- The need to evaluate the true idiopathic cases to be differentiated from patients with secondary asthenozoospermia, to understand the real value of this flow cytometry parameter in the clinical practice;
- The difference between this study (which may seem confirmatory) with others in the literature who have not demonstrated this aspect on this specific category of patients (as mentioned above).

## 6. Conclusions

In conclusion, the results of this study showed a relationship between MMP values after semen collection and sperm motility decline over time. We suggest to use this test to predict a sperm motility curve and, hence, to better evaluate sperm fertilizing capability especially in ART cycles. Even in the context of idiopathic male infertility and for the best individualization of antioxidant therapy (e.g., prokinetics), this flow cytometric parameter could be helpful.

## Figures and Tables

**Table 1:** Sperm parameters (mean ± SEM) of the two groups.

Sperm Parameters	Values (Group A)	Values (Group B)
Volume (mL)	2.7 ± 0.5	3.5 ± 0.7
Sperm concentration (million/mL)	47.9 ± 3.5	54.7 ± 5.2
Total sperm count (million/ejaculate)	170 ± 20.6	158 ± 37.5
Normal forms (%)	5.0 ± 0.8	3.9 ± 0.5
Leukocyte concentration (million/mL)	0.5 ± 0.01	0.5 ± 0.08

**Table 2:** Mitochondrial membrane potential (high, H-MMP, and low, L-MMP) (range) of the patients of groups A and B.

Parameter	Group A (*n* = 18)	Group B (*n* = 13)
Sperm L-MMP (%)	20.0–36.4	37.1–60.1
Sperm H-MMP (%)	63.6–79.1	38.7–59.7
